# Prevalence of Biogenic Amines and Their Relation to the Bacterial Content in Ripened Cheeses on the Retail Market in Poland

**DOI:** 10.3390/foods14142478

**Published:** 2025-07-15

**Authors:** Marzena Pawul-Gruba, Edyta Denis, Tomasz Kiljanek, Jacek Osek

**Affiliations:** 1 Department of Microbiology of Food and Feed, National Veterinary Research Institute, Partyzantów 57, 24-100 Pulawy, Poland; edyta.denis@piwet.pulawy.pl (E.D.); josek@piwet.pulawy.pl (J.O.); 2 Department of Chemical Research of Food and Feed, National Veterinary Research Institute, Partyzantów 57, 24-100 Pulawy, Poland; tomasz.kiljanek@piwet.pulawy.pl

**Keywords:** biogenic amines, ripened cheeses, food analysis, HPLC, MALDI-TOF MS, bacteria

## Abstract

Biogenic amines (BA) are simple organic bases of low molecular weight, formed during decarboxylation of amino acids. Ripened cheeses provide suitable conditions for the development of bacteria and production of BAs. The aim of the present study was to investigate the presence of eight BAs in ripened cheese samples (n = 125) using a high-performance liquid chromatography with diode array detector (HPLC-DAD). Furthermore, microbiological analyses towards identification of bacteria using matrix-assisted laser desorption ionisation—time of flight mass spectrometry (MALDI-TOF MS) were performed. Cadaverine and putrescine were detected in 28.0% and 20.8% of cheese samples at concentrations ranging from 6.12 to 2871 mg/kg and 5.74 to 441 mg/kg, respectively. High amounts of putrescine and cadaverine in cheeses were associated with the presence of Hafnia alvei. Tyramine was identified in 28.0% of samples in the concentration range of 5.62–646 mg/kg. High concentrations of this amine was found in cheeses containing Enterococcus faecium and Enterococcus faecalis. Histamine content, the only BA restricted in food according to Regulation 2073/2005, was observed above 100 mg/kg in 11.2% of the cheeses. Ripened cheeses available on the local retail market may contain significant levels of biogenic amines and may pose a potential health hazard to consumers.

## 1. Introduction

Biogenic amines (BAs) are defined as organic bases of low molecular weight with aliphatic, heterocyclic, or aromatic structures [1]. These biologically active compounds are mainly produced by microbial decarboxylation of amino acids or amination and transamination of aldehydes and ketones [2]. Among the most important biogenic amines found in foods are histamine, tyramine, putrescine, cadaverine, 2-phenylethylamine, tryptamine, spermine, and spermidine [3,4]. Due to the number of amino groups and biosynthesis, they are classified as monoamines (tyramine, 2-phenylethylamine), diamines (putrescine, cadaverine, and tryptamine), and polyamines (spermine, spermidine) [1,5]. BAs occur in a wide range of foods, such as fish, cheeses, meat, beer, wine, and other fermented foods [6,7,8]. High amounts of toxic BAs are often detected in ripened foods such as cheeses with high protein concentrations and free amino acids [9].

During ripening, cheese proteins are degraded, resulting in the formation of free amino acids, which can subsequently be transformed into BAs mainly due to the presence of microorganisms possessing the amino acid decarboxylase, such as lactic acid bacteria (LAB), *Enterococcus* spp. and *Enterobacteriaceae* [10,11]. This process is dependent on the quality of the initial raw materials, hygienic conditions maintained during processing, as well as technological parameters such as temperature and storage period, pH, water activity, and concentrations of salt and sugar [12,13]. Furthermore, the inclusion of starter cultures may influence the production of biogenic amines through their interaction and competition with nonstarter microorganisms for accessible substrates [14,15]. Consequently, it is important that microbial starter cultures are selected and controlled so that, during the fermentation process, they do not generate BAs of concern [16,17].

Biogenic amines, when present in low concentrations, are necessary for many physiological processes [18,19]. However, in high concentrations, BAs can harm nervous and intestinal systems and increase blood pressure. Histamine and tyramine are considered as the most toxic and food safety-relevant amines [20,21]. Furthermore, putrescine and cadaverine as competitive substrates interfere with the degradation of histamine by the diamine oxidase enzyme, thereby increasing histamine toxicity [22]. These amines may also have carcinogenic properties as precursors of carcinogenic N-nitroso compounds [12,23].

There is a lack of regulatory criteria for permissible levels of biogenic amines in ripened cheeses, probably due to insufficient information on BAs content in this food category. Also in Poland, the data available is limited; therefore, considering the toxicity of BAs, these studies are very important. The European Commission in Regulation 2073/2005 lays down food safety criteria only for histamine in fish and fishery products from fish species associated with a high amount of histidine between 100 and 200 mg/kg [24]. However, a general interest exists in reducing the presence of all BAs in all food products.

The aim of this study was to determine the presence and content of BAs (tryptamine, 2-phenylethylamine, putrescine, cadaverine, histamine, tyramine, spermidine, and spermine) and carry out microbiological analyses of samples of commercially available maturing cheeses in Poland, followed by species-level identification of bacteria, in order to investigate the relationship between the concentration of BAs and the number and species of the bacteria.

## 2. Materials and Methods

### 2.1. Cheese Samples

A total of 125 samples of ripened cheeses were analysed, including mould-ripened soft cheeses such as Brie and Camembert (38 samples), blue-veined cheeses such as Roquefort and Gorgonzola (44 samples), semi-hard cheeses such as Raclette and Breton (14 samples), and hard cheeses such as Grana Padano and Parmesan (29 samples). Samples, regardless of brand, were purchased between January and December 2022 from a retail market in the Pulawy region. These kinds of cheeses are also available throughout Poland. After purchase, the cheeses were immediately delivered to the laboratory at refrigerated (1–8 °C) temperature. Most of the cheeses originated from France, Italy, Spain, and Germany in the retail packages. All cheeses tested were within their expiration date.

### 2.2. Chemicals and Reagents

The BAs’ analytical standards (tryptamine, 2-phenylethylamine, putrescine, cadaverine, histamine, tyramine, spermidine, and spermine), as hydrochloride salts, all 98% or higher purity, were obtained from Sigma-Aldrich (St. Louis, MI, USA) and Acros Organics (Geel, Belgium). The internal standard (IS) 1,7 diaminoheptane, dansyl chloride, and L-proline were purchased from Sigma-Aldrich. HPLC grade acetonitrile and acetone were supplied from Merck (Darmstadt, Germany). Sodium carbonate, perchloric acid, and toluene were obtained from Avantor Performance Materials Poland S.A. (Gliwice, Poland). Water was used for the mobile phase and the standards solution preparation was purified using a Mili-Q Plus water system (Merck Millipore; Billerica, MA, USA).

### 2.3. Biogenic Amines Determination

Concentrations of the eight BAs (tryptamine, 2-phenylethylamine, putrescine, cadaverine, histamine, tyramine, spermidine, and spermine) in cheese samples were analysed using HPLC-DAD, according to the analytical method described by Pawul-Gruba et al. [25]. Briefly, a 5 g of homogenised sample enriched with IS was extracted with 10 mL of 0.2 M perchloric acid (HClO_4_) and subjected to centrifugation. Subsequently, 100 μL of supernatant was mixed with 200 μL of saturated sodium carbonate and derivatized with 400 μL of dansyl chloride (7.5 mg/mL) in a heating module at 60 °C for 15 min in the absence of light. In the next step, 100 μL L-proline (10 mg/mL) was added and stored in the dark for an additional 15 min. Further, 500 μL toluene was added, shaken, and maintained for 20 min at ≤−18 °C in order to freeze the aqueous phase. Afterwards, toluene phase was recovered and evaporated under nitrogen at 44 °C and residue was reconstituted in 200 μL of acetonitrile.

The chromatographic analysis was conducted using HPLC-DAD Varian Model 330 Pro Star (Varian, Eindhoven, the Netherlands) equipped chromatographic column Unisol C18 150 × 4.6 mm, 3 μm and precolumn 10 × 3 mm, 3 μm (Agela Technologies, Torrance, CA, USA) with the detection wavelength of 215 nm. The Galaxie Workstation software version 1.7 (Varian) was employed for data analysis. A 10 μL sample was injected. The column temperature was 30 °C, flow rate was 1 mL/min and total analysis run time was 30 min. The mobile phase was composed of acetonitrile (A) and water (B). The gradient programme was performed as follows: initial 60% acetonitrile content increased to 75% in 6 min and hold 2 min, increased to 95% in 5 min and hold 7 min, decreased to 60% in 1 min and hold with 9 min for re-equilibration as described by Pawul-Gruba et al. [25].

The quantitative analyses were conducted by an internal standard method, determining peak heights. Calibration graphs were created through preparing the standard solutions of eight BAs within the range of 5–200 mg/kg of each amine. The limit of detection (LOD) was calculated between 1.53 and 1.88 mg/kg, and the limit of quantification (LOQ) varied from 5.13 to 6.28 mg/kg. The relative standard deviations for the inter-day precision were between 4.1% (cadaverine) and 13.5% (spermine). The intra-day precision ranged from 0.7% (tyramine) to 3.0% (putrescine). The recovery values for BAs were within the range of 74.5% (spermine)–100.2% (cadaverine) [25].

### 2.4. Microbiological Analyses and Identification of Bacteria

For microbiological analyses, 10 g of cheese sample was diluted in 90 mL of dipotassium hydrogenphosphate solution (Chempur, Piekary Śląskie, Poland) and homogenised. Then, the serial decimal dilutions were prepared to determine the total number of microorganisms, the number of mesophilic lactic acid bacteria, *Enterobacteriaceae* and *Enterococcus* spp. The total number of microorganisms was determined according to the ISO 4833-1 Standard [26] on a plate count agar (PCA, BTL, Łódź, Poland). After 72 h of incubation at 30 °C, bacterial colonies were enumerated on the plates containing up to 300 colonies. The number of mesophilic lactic acid bacteria was determined according to the ISO 15214 Standard [27] on de Man–Ragosa–Sharpe agar (Becton, Dickinson and Company, Franklin Lakes, NJ, USA). After 72 h of incubation at 30 °C, typical bacterial colonies were enumerated on the plates containing up to 300 colonies. The number of *Enterobacteriaceae* was determined according to the ISO 21528-2 Standard [28] on violet-red bile agar with glucose (VRBG, Bio-Rad, Marnes-La-Coquette, France) by incubation at 37 °C for 24 h. Typical bacterial colonies were enumerated on the plates containing up to 150 colonies. Confirmatory oxidase and glucose fermentation tests were performed. The number of *Enterococcus* spp. was determined on Slanetz and Bartley agar (Thermo Fisher Scientific, Waltham, MA, USA) by incubation at 37 °C for 48 h. Typical bacterial colonies were enumerated on the plates containing up to 150 colonies. The results of enumeration of bacteria were presented as logarithmic values of the number of colonies per gram of cheese (log_10_ CFU/g). Subsequently, up to five bacterial colonies grown on de Man–Ragosa–Sharpe agar, VRBG, and Slanetz and Bartley agar were selected from the highest sample dilutions to determine the bacterial species.

Identification of bacterial species was performed using matrix-assisted laser desorption ionisation—time of flight mass spectrometry (MALDI-TOF MS) (Bruker, Bremen, Germany). Bacterial colonies grown on non-selective media were transferred into a 1.5 mL centrifuge tube containing 300 μL deionized water and mixed using a vortex. Subsequently, 900 μL ethanol (min. 99.8%) (J.T. Baker, Gdańsk, Poland) was added and centrifuged at 14.000 rpm for 2 min and the supernatant was decanted. After re-centrifugation, the supernatant was discarded by pipetting, and the pellet was dried for 5–10 min. Then, 40 μL of 70% formic acid (Honeywell—Fluka, Seelze, Germany) and 40 μL acetonitrile (J.T. Baker) were added, mixed, and centrifuged. Further, 1 μL of the supernatant was applied to the plate, evaporated, and covered with 1 μL of matrix solution (α-cyano-4-hydroxycinnamic acid, 10 mg/mL). All samples were applied into the plate in duplicates. Instrumental analysis was conducted according to the recommendations of the Biotyper system producer using MBT Compass 4.1.70 software and compatible spectral libraries (Bruker).

### 2.5. Statistical Analysis

Statistical analysis of data for dependencies between the level of determined of BAs and the number and species of identified bacteria was performed using GraphPad Prism 10 software (Boston, MA, USA). For this purpose, Spearman correlation was used by determining correlation coefficients (r_s_), which ranges between −1 and 1, r < 0 indicates a negative correlation (red), r > 0 indicates a positive correlation (blue). Spearman correlation is considered statistically significant if the *p* value is less than the significance level (alpha = 0.05) and *, **, *** and **** represent *p* < 0.05, *p* < 0.01, *p* < 0.001, and *p* < 0.0001, respectively.

## 3. Results and Discussion

### 3.1. Biogenic Amines in Ripened Cheeses

A total of 125 different types of ripened cheeses (mould-ripened, blue-veined, semi-hard, and hard cheeses) were investigated towards the presence of BAs. At least one biogenic amine was identified in 97 (77.6%) of samples in a concentration above LOQ. One biogenic amine was detected in 40 (32.0%) samples, two BAs in 33 (26.4%) samples, three BAs in 16 (12.8%) samples, and a maximum four BAs were found in 8 (6.4%) samples. Table 1, Table 2 and Table 3 and the Supplementary Table S1 show concentrations of the BAs determined in ripened cheeses. The statistical assessment for all biogenic amines is presented in Table 4. Differences among the BAs were observed not only in relation to the kind of cheese (mould-ripened, blue-veined, semi-hard, and hard) but also among samples within the same type. As shown by other authors, BAs content may depend not only on the type of cheese, but also on the production process, which is related to the content of bacteria in the milk, cheese ripening length, and ripening and storage temperature [14,29]. The main amines identified in ripened cheeses were histamine, tyramine, cadaverine, and putrescine.

Histamine was found in 35 (28.0%) samples at levels between 6.23 and 342 mg/kg. The maximum level of histamine was detected in Gorgonzola (342, 298, 246, and 194 mg/kg) and in Parmigiano Reggiano (163 mg/kg). The EFSA concluded that no adverse health consequences are observed after exposure to 50 mg of histamine (per person per meal) for healthy individuals, but below detectable limits for people with histamine intolerance [16]. Thus, considering the maximum detected histamine concentration (342 mg/kg) and a normal portion of cheese of 30 g, a total ingestion of 10.3 mg is below the limit proposed by EFSA for healthy individuals, but for persons with histamine intolerance it may pose a potential risk [16]. Fourteen (11.2%) samples of the ripened cheeses, mainly Gorgonzola, Parmigiano Reggiano and Grana Padano contained histamine above 100 mg/kg. According to the European Union regulation on the microbiological criteria for foodstuffs, 100 mg/kg is the maximum limit of histamine for some fish and fish products [24]. A similar result was reported by other authors, where content of histamine above 100 mg/kg was found in 13.8% of samples and the highest concentrations up to 1159 mg/kg were determined in samples of hard regional cheeses and up to 255 mg/kg in Gorgonzola [11]. In a study performed in Spain, histamine was identified in 51.2% of cheese samples, with the concentration ranging from 5 to 571 mg/kg, and in 33% of the samples, histamine contents were above 100 mg/kg. The highest concentrations of histamine, up to 500 mg/kg, were determined in hard cheeses [30].

Tyramine was detected in 35 (28.0%) cheese samples with the concentration range of 5.62–646 mg/kg. The highest content was found in three samples of Gorgonzola (646, 583 and 411 mg/kg) and in Morbier (334 mg/kg) cheeses. The content of tyramine above 100 mg/kg was found in 13 (10.4%) samples. According to the EFSA scientific opinion, exposure to a level of 600 mg of tyramine in food (per person per meal) for healthy individuals, 50 mg for those taking the third generation of monoamino oxidase inhibitor (MAOI) drugs, and 6 mg for those taking of classical MAOI drugs does not cause any negative health effects [16]. Thus, considering the maximum tyramine concentration detected (646 mg/kg) in blue-veined cheeses and a normal portion of cheese of 30 g per person, a total ingestion would amount to 19.4 mg of tyramine, which is below the limit proposed by EFSA for healthy individuals and those taking the third generation of MAOI drugs, but may pose a potential risk to individuals taking classic MAOI [16]. In the case of the maximum detected tyramine concentrations among semi-hard cheeses up to 334 mg/kg (10.02 mg/30 g) and hard cheeses up to 262 mg/kg (7.86 mg/30 g), the estimated level of 6 mg per meal [16] for people with classical MAOI medication was exceeded, which may also pose a health risk to this very sensitive group of consumers. MAOI restrain the activity of enzymes that cause the breakdown of biogenic amines, which can lead to the accumulation of large amounts of BAs in the body [31]. Similar results were obtained in our previous study, where the tyramine was detected in 28.6% of samples in the concentration range of 7.28–692 mg/kg [25]. A high content of tyramine in mould-ripened cheese (763 mg/kg) and semi-hard cheese (767 mg/kg) were also reported by Zdolec et al. [32].

Cadaverine was detected in 35 (28.0%) samples at concentrations between 6.12 and 2871 mg/kg and putrescine was found in 26 (20.8%), with a concentration range from 5.74 to 441 mg/kg. The cadaverine and putrescine levels above 100 mg/kg were identified in 13 (10.4%) and 7 (5.6%) samples, respectively. The maximum levels of cadaverine were found in samples of Camembert (2871, 1802, 829, and 633 mg/kg, respectively), in Brie (1137 mg/kg), in Coulommiers (828 mg/kg), and in semi-hard cheese Morbier (506 mg/kg). The highest content of putrescine was detected in the same samples as those with cadaverine, i.e., in Camembert (441 mg/kg), Brie (331 mg/kg), and Morbier (304 mg/kg). As found in another survey, the maximum levels of cadaverine (748 mg/kg) and putrescine (523 mg/kg) were detected in the soft-ripened cheese Olmützer Quargel [11].

2-phenylethylamine was identified at lower level than the amines previously described. This amine was detected in 8 (6.4%) samples in the concentration range of 8.37–36.1 mg/kg. The highest concentration of 2-phenylethylamine was found in the semi-hard cheese Raclette (36.1 mg/kg). Other authors reported that 2-phenylethylamine concentrations in cheese samples produced from pasteurised milk was up to 53 mg/kg [33].

Spermidine was found in the concentration ranged from 6.43 mg/kg to 21.3 mg/kg in 45 (36.0%) samples. Similar results for this amine up to 37.6 mg/kg (Grana Padano) was obtained by Mayer et al. [11]. Spermine was detected in only two samples at levels of 6.52 mg/kg and 7.28 mg/kg, respectively. On the other hand, tryptamine was not found in any sample tested. However, in studies conducted in Spain, this amine was detected in 16% of cheese samples where the mean concentration was 29.21 mg/kg [34].

**Figure 1:**
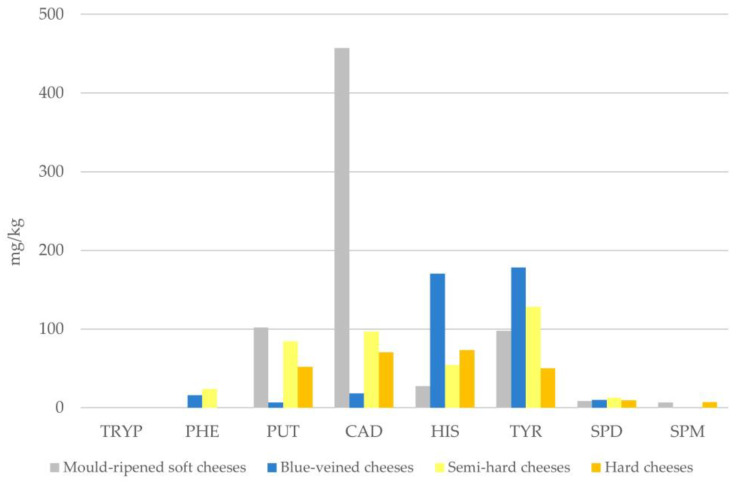
Mean values of BAs content in positive samples of ripened cheeses. TRYP: tryptamine, PHE: 2-phenylethylamine, PUT: putrescine, CAD: cadaverine, HIS: histamine, TYR: tyramine, SPD: spermidine, SPM: spermine.

The present results showed that cheeses available in the market may contain significant amounts of biogenic amines. Among the analysed ripened cheeses, the highest average concentrations of histamine were found in blue-veined and hard cheeses, tyramine in blue-veined and semi-hard cheeses, while putrescine and cadaverine in mould-ripened and semi-hard cheeses (Figure 1). These results are consistent with those of other studies [11,30,32]. Taking into account reports on synergistic actions between BAs [22], which may enhance the adverse effects of histamine on humans, attention should be paid to Morbier and Camembert cheeses which contained histamine, putrescine, cadaverine, and tyramine at the same sample (total concentrations 1256 and 831 mg/kg, respectively). Furthermore, because tyramine can also create a synergistic toxic effect with histamine [35], two Gorgonzola cheeses (total concentrations 912 and 504 mg/kg, respectively) containing both of these amines may pose a potential health hazard.

### 3.2. Microbiological Analysis and Identification of Bacterial Species

Results of microbiological analyses of mould-ripened, blue-veined, and semi-hard/hard cheeses are shown in Table 5. Lactic acid bacteria (LAB) were the most numerous microorganisms in all types of cheeses tested. They were present in 96.8% of all investigated samples in the range from 2.88 to 9.34 log_10_ CFU/g (Table 5). The highest number of LAB was identified in the semi-hard cheese Raclette (9.34 log_10_ CFU/g) and the mould-ripened cheese Chevre St. Maure (9.26 log_10_ CFU/g). In the blue-veined cheeses, there was a problem with the isolation of lactic acid bacteria because the mould grew over the entire plate, but due to the transparency of the medium, it was possible to enumerate bacterial colonies that even grew under mould. These were probably *Penicillium glaucum* and *Penicillium roqueforti* moulds, which are used in the production of cheeses such as Gorgonzola, Roquefort, and Fourme d’Ambert.

*Enterococcus* spp. belongs to LAB but due to the methodology used to determine their number, they were analysed as a separate group. *Enterococcus* spp. was detected in 48 (38.4%) of all tested samples in the range from 2.00 to 7.60 log_10_ CFU/g. In the blue-veined cheese, these bacteria were found in 47.7%, in mould-ripened cheese in 44.7%, in the semi-hard cheeses in 42.9%, and in the hard cheeses in 13.8% of samples, respectively. The highest number of enterococci was identified in the Gorgonzola (7.60 and 7.51 log_10_ CFU/g) and Camembert (7.32 and 7.20 log_10_ CFU/g) cheeses.

Among a total of 125 samples tested, *Enterobacteriaceae* bacteria were found in 30 (24.0%) of cheese samples in the range of 1.00–7.86 log_10_ CFU/g. These microorganisms were mainly present in mould-ripened (55.3%) and semi-hard (28.6%) cheeses. The maximum number of *Enterobacteriaceae* was identified in three samples of Camembert (7.86, 7.85 and 7.78 log_10_ CFU/g).

The cheese samples positive for *Enterobacteriaceae*, *Enterococcus* spp. or LAB were further examined to determine the bacterial species using MALDI-TOF MS (Table 1, Table 2 and Table 3). Among *Enterobacteriaceae*, the most common species was *Hafnia alvei* identified mainly in the mould-ripened and semi-hard cheeses. The less frequently found bacteria were *Raoultella ornithinolytica* and *Lelliottia amnigena* in samples of mould-ripened cheese and *Serratia liquefaciens* in the blue-veined and semi-hard cheeses.

Among *Enterococcus* spp., the most dominant species were *Enterococcus faecalis* and *Enterococcus faecium*. These two bacterial species were most often identified in blue-veined and mould-ripened cheeses. Other bacteria such as *Enterococcus malodoratus* and *Enterococcus hirae* were detected to a lesser extent.

Among the LAB, the most frequently identified bacterial species were *Lactococcus lactis*, *Lacticaseibacillus paracasei*, *Leuconostoc mesenteroides*, and *Leuconostoc pseudomesenteroides*. *Lactococcus lactis* was most abundant in mould-ripened and semi-hard cheeses. Most *Lacticaseibacillus paracasei* was detected in the hard cheeses, whereas *Leuconostoc mesenteroides* and *Leuconostoc pseudomesenteroides* were most frequently isolated from the blue-veined and mould-ripened cheeses. Less frequently identified species of LAB were *Lacticaseibacillus rhamnosus*, *Lactobacillus curvatus*, *Pediococcus acidilactici*, *Lactobacillus plantarum*, and *Lactobacillus delbruecki*.

In the blue-veined cheeses, a yeast of the family *Saccharomycetaceae Debaryomyces hansenii* and *Saccharomyces cerevisiae* were also found.

### 3.3. Correlation Between Biogenic Amines and the Presence of Bacteria

**Figure 2:**
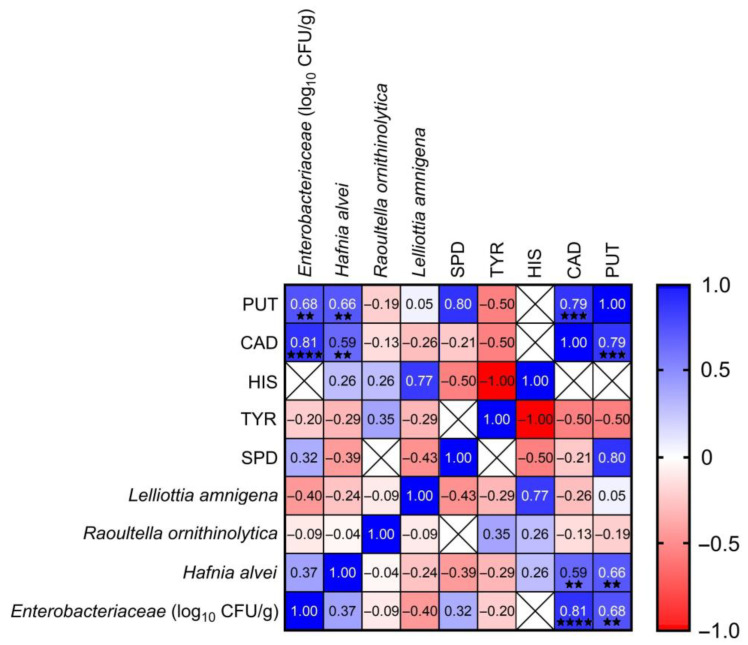
Correlation between *Enterobacteriaceae* and biogenic amines in the mould-ripened cheese (*n* = 38). PUT: putrescine, CAD: cadaverine, HIS: histamine, TYR: tyramine, SPD: spermidine. The Spearman correlation coefficient r ranges from −1 to 1, r < 0 indicates a negative correlation (blue), r > 0 indicates a positive correlation (red), *p* value: ^★^^★^ < 0.01, ^★^^★^^★^ < 0.001, and ^★^^★^^★^^★^ < 0.0001.

The relationship between the concentration of BAs and the number and identified bacterial species was determined with the Spearman correlation coefficient (r_s_). The results showed that *Enterobacteriaceae* positively correlated with putrescine (r = 0.54) and cadaverine (r = 0.76), considering all samples tested (*n* = 125). In mould-ripened cheeses *Enterobacteriaceae* statistically significantly positively correlated with putrescine (r = 0.68) and cadaverine (r = 0.81) (Figure 2). Among the identified bacterial species, *Hafnia alvei* correlated positively with putrescine (r = 0.66) and cadaverine (r = 0.59) (Figure 2). These bacteria were detected in 15 samples, which is 39.5% of all mould-ripened cheeses tested. The concentrations of putrescine and cadaverine in these cheese samples were up to 441 mg/kg and 2871 mg/kg, respectively. Furthermore, in mould-ripened cheese samples, putrescine was often detected together with cadaverine, which resulted in a statistically significantly positive correlation of r = 0.79 between these two amines (Figure 2). Similar results were described by other authors who studied the relationship of BAs with bacteria isolated from cheeses, meat, and fermented sausages. They found that the most of the *Enterobacteriaceae* isolates (147 of 149) formed either putrescine or cadaverine and the most commonly isolated species were *Hafnia alvei* and *Serratia liquefaciens* [36]. *Enterobacteriaceae* identified in cheeses are responsible for the production of putrescine [37]. Zdolec et al. [32] also showed that these bacteria in the cheeses correlated positively with putrescine (r = 0.81).

In the present study, among the total samples tested, *Enterococcus* spp. was statistically significantly positively correlated (r = 0.47) with tyramine. In the mould-ripened and blue-veined cheeses *Enterococcus* ssp. correlated with tyramine (r = 0.60 and r = 0.45, respectively), but it was not statistically significant, probably due to the small number of pairs formed. The most frequently identified bacterial species in these types of cheeses were *Enterococcus faecalis* and *Enterococcus faecium*. In the samples containing these bacteria, also a high level of tyramine was detected, such as 156 mg/kg (Camembert Petit Normand) and 411 mg/kg, 360 mg/kg (Gorgonzola). In another study, *Enterococcus* spp. isolated from food were also associated with the presence of tyramine. Considering all *Enterococcus* spp. isolates (*n* = 137), *Enterococcus faecalis* and *Enterococcus faecium* were identified in 75 and 62 samples, respectively. In addition, *Enterococcus faecalis* caused tyramine formation in more samples than *Enterococcus faecium* [36]. This is also consistent with our results, where among all samples examined, *Enterococcus faecalis* was detected in 30 samples, while *Enterococcus faecium* was detected in 19 samples. In the studies conducted in Italy, all *Enterococcus faecium* strains derived from Pecorino cheese made from raw ewe’s milk demonstrated the ability to produce tyramine [38].

**Figure 3:**
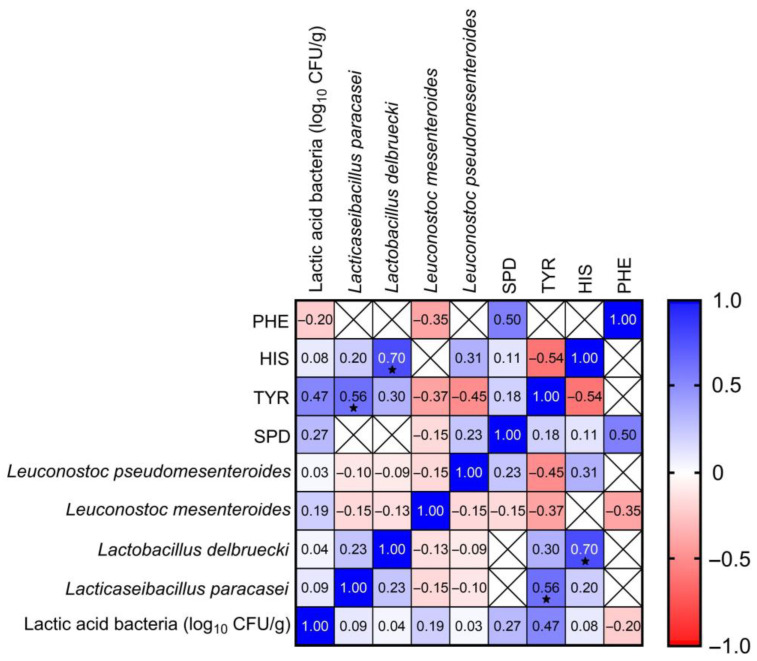
Correlation between lactic acid bacteria and biogenic amines in the blue-veined cheeses (*n* = 44). PHE: 2-phenyloetyloamina, HIS: histamine, TYR: tyramine, SPD: spermidine. The Spearman correlation coefficient r ranges from −1 to 1, r < 0 indicates a negative correlation (blue), r > 0 indicates a positive correlation (red), *p* value: ^★^ < 0.05.

LAB are the main bacteria in the production of cheese [32]. They may be present in raw milk or constitute starter and non-starter cultures [37,39]. In our study, in blue-veined cheeses the number of lactic acid bacteria showed a positive relationship with tyramine (r = 0.47), *Lacticaseibacillus paracasei* was especially statistically significantly positive correlated (r = 0.56) with this biogenic amine (Figure 3). In other studies, in samples with tyramine above 100 mg/L, *Lactobacillus* was detected in 21.6% of the cultured isolates [36]. Ladero et al. [37] reported that tyramine was the most frequently occuring amine in ripening foods in which *Enterococcus* spp. and *Lactobacillus* were most often identified. *Lactobacillus delbruecki* demonstrated the statistically significantly positively correlation with histamine (r = 0.70) in the blue-veined cheeses tested (Figure 3). In three Gorgonzola cheese samples containing this bacterium, the highest histamine concentrations were found (342 mg/kg, 298 mg/kg, and 236 mg/kg). LAB, the bacteria involved in the fermentation process, are considered as the main histamine producers [40].

In hard cheeses such as Parmigiano Reggiano and Grana Padano, the presence of *Lacticaseibacillus paracasei*, *Lacticaseibacillus rhamnosus*, and *Pediococcus acidilactici* were associated with the occurrence of histamine in the concentration range of 23.1–163 mg/kg, but no positive correlations were found. A similar study on the presence of LAB in long-ripened hard cheese (Parmigiano Reggiano) was conducted in Italy. In this cheese matured for up to 24 months, the occurrence of non-starter lactic acid bacteria (NSLAB) of the *Lactobacillus* genus (especially *Lactobacillus paracasei* and *Lactobacillus rhamnosus*) and *Pediococcus* (*Pediococcus acidilactici*) was found, which is also consistent with our results [41]. The NSLAB grow very slowly in milk and practically do not participate in milk acidification. The most common type of NSLAB in cheeses are mesophilic bacteria of the genus *Lactobacillus* [15]. In mould-ripened and semi-hard cheeses, *Lactococcus lactis*, used as starter lactic acid bacteria (SLAB), was identified in the largest numbers. Starter bacteria of the *Lactococcus lactis* species primarily decide the rate of milk souring and lactic acid production [15].

The present results showed that *Enterobacteriaceae* (*Hafnia alvei*) correlated with putrescine and cadaverine, whereas *Enterococcus* spp. with tyramine. The presence of LAB (*Lactobacillus*) in cheese was related to the content of tyramine and histamine. The obtained findings confirm the results of other studies [32,36,38]. Given the high number of microorganisms present in cheeses, it is very difficult to isolate and identify those minority species that could be relevant as BAs producers, such as *Lactobacillus parabuchneri*, a species that, according to several authors, is the main producer of histamine in cheeses [40,42,43].

## 4. Conclusions

A large amount of free amino acids and a long ripening period of cheeses are suitable conditions for the formation of biogenic amines. In the present study, it was shown that 77.6% of cheese samples contained at least one BA. Cadaverine, putrescine, tyramine, and histamine were quantitatively the most common biogenic amines. The presence of cadaverine and putrescine was associated with the presence of *Enterobacteriaceae* (*Hafnia alvei*). High concentrations of tyramine were found in ripened cheeses when *Enterococcus faecium* and *Enterococcus faecalis* were present. The content of histamine above the limit of 100 mg/kg were found in 11.2% of the ripened cheeses, mainly Gorgonzola, Parmigiano Reggiano, and Grana Padano. The long ripening period of cheeses contributes to the formation of these biogenic amines. Taking into account the type of cheese (mould-ripened, blue-ripened, semi-hard, and hard), differences in the correlations between the identified bacteria and amines were observed. Based on the conducted studies, it was found that ripened cheeses available on the retail market may contain significant amounts of biogenic amines. Considering the risk analysis of negative health effects after consuming BAs in cheeses, it was found that histamine and tyramine do not pose a health risk to healthy people. However, in the case of persons with histamine intolerance or those taking drugs belonging to the classic monoamine oxidase inhibitors, they may pose a potential health risk. This group of sensitive consumers is not recommended to eat ripened cheeses, especially blue-veined cheeses, in which the highest amounts of histamine and tyramine were found. The obtained results and the growing consumer interest in the ripened cheeses subject to this study indicate the need to establish an acceptable level for biogenic amines and to conduct further research on the safety of this type of food category.

## Figures and Tables

**Table 1:** Biogenic amines concentration ranges (TRYP—tryptamine, PHE—2-phenylethylamine, PUT—putrescine, CAD—cadaverine, HIS—histamine, TYR—tyramine, SPD—spermidine, SPM—spermine) and main microorganisms identified in mould-ripened soft cheeses.

Samples	No. *	Biogenic Amines Concentration Ranges (mg/kg)								Main Microorganisms Identified
		TRYP	PHE	PUT	CAD	HIS	TYR	SPD	SPM	
Mould-ripened soft cheeses (*n* = 38)										
Camembert	12	-	-	7.76–441	8.23–2871	21.9	156	6.64–10.3	-	- *Hafnia alvei* - *Enterococcus faecalis* - *Lactococcus lactis* - *Leuconostoc pseudomesenteroides* - *Lactobacillus plantarum*
Brie	5	-	-	6.45–331	10.8–1137	-	9.09	8.29	-	- *Hafnia alvei* - *Raoultella ornithinolytica* - *Enterococcus faecalis* - *Enterococcus malodoratus* - *Lactobacillus plantarum* - *Lacticaseibacillus paracasei* - *Lactococcus lactis*
Munster	3	-	-	37.7	8.81	9.43–68.5	9.19–186	6.48–9.62	-	- *Lelliottia amnigena* - *Enterococcus faecalis* - *Enterococcus faecium* - *Lactococcus lactis*
La Brique	2	-	-	-	70.6	-	-	-	-	- *Hafnia alvei* - *Lactococcus lactis*
Snack	2	-	-	-	-	-	-	-	-	- *Lelliottia amnigena* - *Enterococcus glivus*
Soft cheese	2	-	-	-	7.12	-	-	7.66–8.66	-	- *Serratia liquefaciens* - *Lactococcus lactis* - *Leuconostoc pseudomesenteroides*
Other **	12	-	-	19.0–110	14.8–828	10.7	-	8.96–11.9	6.52	- *Hafnia alvei* - *Enterococcus faecalis* - *Enterococcus faecium* - *Lactococcus lactis* - *Leuconostoc mesenteroides* - *Leuconostoc pseudomesenteroides*

*—number of samples. **—one of each: Caprice des Dieux, Carre de l’Est, Chevre St. Maure, Coulommiers, Fromage de Bretange, Fromage de Normandie, Fromage du Calvados, La Rustique Carre, Langres, Pave d’Affiniois, Saint Albray, and Saint Felicien.

**Table 2:** Biogenic amines concentration ranges (TRYP—tryptamine, PHE—2-phenylethylamine, PUT—putrescine, CAD—cadaverine, HIS—histamine, TYR—tyramine, SPD—spermidine, SPM—spermine) and main microorganisms identified in blue-veined cheeses.

Samples	No. *	Biogenic Amines Concentration Ranges (mg/kg)								Main Microorganisms Identified
		TRYP	PHE	PUT	CAD	HIS	TYR	SPD	SPM	
Blue-veined cheeses (*n* = 44)										
Gorgonzola	15	-	8.37–35.8	7.57	30.0	6.23–342	5.88–646	7.20–9.39	-	- *Serratia liquefaciens* *- Enterococcus faecium* *Lactobacillus delbruecki* *Lacticaseibacillus paracasei*
Roquefort	5	-	-	-	6.12	-	21.6–101	6.97–8.76	-	*Enterococcus hirae* *Enterococcus faecalis* *Enterococcus faecium* *Leuconostoc mesenteroides*
Fourme d’Ambert	4	-	15.7	-	-	-	-	10.1–13.8	-	*Enterococcus faecalis* *Leuconostoc mesenteroides* *Leuconostoc pseudomesenteroides*
Bleu d’Auvergne	3	-	-	-	-	-	-	6.43–9.52	-	*Leuconostoc mesenteroides*
Danish Blue Cheese	3	-	11.1	-	-	-	-	8.06	8.37	*Leuconostoc mesenteroides*
Lazur	3	-	-	-	-	-	-	11.1–14.8	-	*Leuconostoc mesenteroides*
Bavaria blue	2	-	9.76	-	-	-	16.8	6.67	-	*Enterococcus faecium* *Enterococcus faecium* *Enterococcus malodoratus* *Leuconostoc mesenteroides*
Cambozola	2	-	-	-	-	-	-	-	-	*Lacticaseibacillus paracasei*
Kamienio-górski	2	-	-	-	-	-	101–133	16.5	-	*Enterococcus faecalis* *Lactobacillus curvatus*
Other **	5	-	-	5.74	-	-	-	6.91–13.9	-	*Enterococcus faecalis* *Leuconostoc mesenteroides* *Leuconostoc pseudomesenteroides Lactobacillus plantarum*

*—number of samples. **—One of each: Bleu des Causses, Dorblu, Le Bleu, Saint Agur, and Stilton.

**Table 3:** Biogenic amines concentration ranges (TRYP—tryptamine, PHE—2-phenylethylamine, PUT—putrescine, CAD—cadaverine, HIS—histamine, TYR—tyramine, SPD—spermidine, SPM—spermine) and main microorganisms identified in semi-hard and hard cheeses.

Samples	No. *	Biogenic Amines Concentration Ranges (mg/kg)								Main Microorganisms Identified
		TRYP	PHE	PUT	CAD	HIS	TYR	SPD	SPM	
Semi-hard cheeses (*n* = 14)										
Raclette	4	-	19.7–36.1	-	7.04–18.4	7.14–68.5	32.2–223	6.58	-	- *Enterococcus malodoratus* - *Enterococcus faecalis* - *Lactococcus lactis*
Tomme de Montagne	2	-	-	7.06	22.3	-	-	-	-	- *Lactococcus lactis*
Other **	8	-	16.1	5.87–304	19.9–506	30.7–112	32.3–334	9.93–21.3	-	- *Hafnia alvei* - *Serratia liquefaciens* - *Enterococcus faecalis* - *Enterococcus faecium* - *Lactococcus lactis* - *Lacticaseibacillus paracasei* - *Lactobacillus curvatus*
Hard cheeses (*n* = 29)										
Parmigiano Reggiano	7	-	-	-	-	53.0–163	6.99–11.4	-	-	- *Lacticaseibacillus paracasei* - *Pediococcus acidilactici*
Grana Padano	6	-	-	-	-	23.1–152	5.62	-	-	- *Lacticaseibacillus rhamnosus* - *Lacticaseibacillus paracasei* - *Pediococcus acidilactici*
Cheddar	2	-	-	-	-	-	-	-	-	- *Lacticaseibacillus paracasei* - *Lacticaseibacillus rhamnosus* - *Lactococcus lactis*
Grated dehydrated cheese	2	-	-	22.3–117	138–180	87.1–91.8	29.9–262	-	-	- *Enterococcus faecium* - *Lacticaseibacillus paracasei* - *Pediococcus acidilactici*
Other ***	12	-	-	17.0	9.82–68.2	8.24	5.62–81.6	6.52–12.4	7.28	- *Hafnia alvei* - *Enterococcus faecalis* - *Lacticaseibacillus paracasei* - *Lactococcus lactis*

*—number of samples. **—One of each: Breton, Caciotta with chilli and arugula, Memel with basil, Mimolette, Morbier, Queijo Curado Mistura, Spanish Matured Goats cheese, and Tricolore. ***—One of each: Formagia, Corregio, Graviera Kritis, Gruyere, Monte Veronese, Old Amsterdam, Oscypek, Pecorino Romano, Queso de Cabra, Queso de Manchego, Queso Iberico, and Scharfer Paul.

**Table 4:** Statistical assessment for positive samples of BAs tested in ripened cheese samples.

	Biogenic Amines Concentrations (mg/kg)								
	TRYP	PHE	PUT	CAD	HIS	TYR	SPD	SPM	Total
Mould-ripened soft cheeses (*n* = 38)									
Minimum	-	-	6.06	6.37	9.43	9.09	6.48	6.52	6.06
Mean	-	-	102	457	27.6	97.7	8.75	6.52	192
Median	-	-	19.0	70.6	16.3	128	8.66	6.52	11.9
90 Percentile	-	-	371	1137	54.5	174	11.1	6.52	499
95 Percentile	-	-	434	1802	61.5	180	11.7	6.52	829
Maximum	-	-	441	2871	68.5	186	11.9	6.52	2871
Pct. of pos. Samples *	0.0%	0.0%	44.7%	55.3%	10.5%	13.2%	39.5%	2.6%	78.9%
Blue-veined cheeses (*n* = 44)									
Minimum	-	8.37	5.74	6.12	6.23	5.88	6.43	-	5.74
Mean	-	16.1	6.66	18.1	171	178	9.96	-	83.3
Median	-	11.1	6.66	18.1	153	61.7	9.18	-	13.9
90 Percentile	-	27.8	7.39	27.6	293	531	14.5	-	256
95 Percentile	-	31.8	7.48	28.8	318	605	15.0	-	365
Maximum	-	35.8	7.57	30.0	342	646	16.5	-	646
Pct. of pos. samples	0.0%	11.4%	4.5%	4.5%	27.3%	31.8%	54.5%	0.0%	81.8%
Semi-hard cheeses (*n* = 14)									
Minimum	-	16.1	5.87	7.04	7.14	32.2	6.58	-	5.87
Mean	-	24.0	84.6	97.0	54.6	128	12.8	-	77.1
Median	-	19.7	14.3	19.2	49.6	89.3	11.6	-	22.0
90 Percentile	-	32.8	219	264	99.0	267	18.9	-	247
95 Percentile	-	34.5	262	385	105	301	20.1	-	324
Maximum	-	36.1	304	506	112	334	21.3	-	506
Pct. of pos. samples	0.0%	21.4%	28.6%	42.9%	28.6%	50.0%	28.6%	0.0%	78.6%
Hard cheeses (*n* = 29)									
Minimum	-	-	17.0	9.82	8.24	5.62	6.52	7.28	5.62
Mean	-	-	52.1	70.6	73.4	50.3	9.46	7.28	60.0
Median	-	-	22.3	42.6	65.1	14.2	9.46	7.28	36.2
90 Percentile	-	-	98.1	159	146	118	11.8	7.28	145
95 Percentile	-	-	108	170	155	190	12.1	7.28	167
Maximum	-	-	117	180	163	262	12.4	7.28	262
Pct. of pos. samples	0.0%	0.0%	10.3%	20.7%	51.7%	31.0%	6.9%	3.4%	69.0%

*—Percentage of positive samples.

**Table 5:** Bacterial counts in different types of ripened cheeses (log_10_ CFU/g).

Microorganisms	Mould-Ripened Soft Cheeses (*n* = 38)		Blue-Veined Cheeses (*n* = 44)		Semi-Hard/Hard Cheeses (*n* = 43)	
	Mean ± SD	Ranges	Mean ± SD	Ranges	Mean ± SD	Ranges
Number of microorganisms	8.78 ± 0.61	6.90–9.60	8.11 ± 0.58	6.96–9.20	6.38 ± 2.02	2.34–9.34
*Enterobacteriaceae*	5.48 ± 1.85	1.86–7.86	2.75 ± 0.40	2.41–3.32	3.28 ± 1.84	1.00–5.88
*Enterococcus* spp.	4.65 ± 1.60	2.30–7.32	4.51 ± 1.58	2.36–7.60	3.73 ± 1.54	2.00–6.72
Lactic acid bacteria	8.03 ± 0.75	6.04–9.26	7.79 ± 0.56	6.74–8.98	6.34 ± 1.81	2.88–9.34

SD—standard deviation.

## Data Availability

The original contributions presented in this study are included in the article. Further inquiries can be directed to the corresponding author.

## Supplementary Materials

The following supporting information can be downloaded at: https://www.mdpi.com/article/10.3390/foods14142478/s1, Table S1: Biogenic amines contents (TRYP—tryptamine, PHE—2-phenylethylamine, PUT—putrescine, CAD—cadaverine, HIS—histamine, TYR—tyramine, SPD—spermidine, SPM—spermine) and microorganisms identified in all cheeses.

## Abbreviations

BA	Biogenic amines
HPLC-DAD	High performance liquid chromatography with diode array detector
MALDI-TOF MS	Matrix-assisted laser desorption ionisation—time of flight mass spectrometry
LAB	Lactic acid bacteria
NSLAB	Non-starter lactic acid bacteria
