# Supplementary material for: Prevalence of Biogenic Amines and Their Relation to the Bacterial Content in Ripened Cheeses on the Retail Market in Poland
Source: Foods. 2025 Jul 15;14(14):2478. doi: 10.3390/foods14142478 (PMC12294292; doi:10.3390/foods14142478)
Supplement: Supplementary file 1 [file foods-14-02478-s001.zip › foods-3709681-supplementary.pdf]

foods

MDPI

Table S1. Biogenic amines contents (TRYP – tryptamine, PHE – 2-phenylethylamine, PUT – putrescine, CAD – cadaverine, HIS – histamine, TYR – tyramine, SPD – spermidine, SPM - spermine) and microorganisms identified in all cheeses.

Samples	Origin	Biogenic amines concentrations (mg/kg)									Identified microorganisms	Total*
		TRYP	PHE	PUT	CAD	HIS	TYR	SPD	SPM	Total		
Mould-ripened soft cheeses (n = 38)												
Camembert	France	-	-	98.2	633	-	-	-	-	731	*Hafnia alvei*<br>*Enterococcus faecalis*<br>*Leuconostoc mesenteroides*<br>*Lactococcus lactis*	8.78
Camembert	France	-	-	441	1,802	-	-	-	-	2,243	*Hafnia alvei*	8.54
Camembert	France	-	-	7.76	268	-	-	6.64	-	282	*Hafnia alvei*<br>*Enterococcus faecalis*<br>*Lactococcus lactis*<br>*Leuconostoc pseudomesenteroides*	9.18
Camembert	Poland	-	-	-	-	-	-	-	-	-	*Lactococcus lactis*<br>*Leuconostoc pseudomesenteroides*	9.00
Camembert	France	-	-	-	-	-	-	10.3	-	10.3	*Lactococcus lactis*	8.98
Camembert	France	-	-	-	-	-	-	-	-	-	*Lactococcus lactis*<br>*Leuconostoc pseudomesenteroides*	8.97
Camembert	France	-	-	14.6	202	-	-	7.14	-	224	*Hafnia alvei*<br>*Enterococcus faecalis*<br>*Lactococcus lactis*<br>*Leuconostoc pseudomesenteroides*	8.89
Camembert	France	-	-	10.2	60.8	-	-	-	-	71.0	*Hafnia alvei*<br>*Enterococcus faecalis*<br>*Lactococcus lactis*	8.90
Camembert Le Caratere	France	-	-	52.4	829	-	-	-	-	881	*Hafnia alvei*<br>*Enterococcus durans*<br>*Lactococcus lactis*	8.32
Camembert Le Rustique	France	-	-	432	2871	-	-	-	-	3,303	*Hafnia alvei*<br>*Leuconostoc mesenteroides*	9.04
Camembert Liqueil	France	-	-	11.4	8.23	-	-	7.39	-	27.0	*Hafnia alvei*<br>*Enterococcus faecalis*<br>*Enterococcus durans*<br>*Lactococcus lactis*	6.90
Camembert Petit Normand	France	-	-	140	513	21.9	156	-	-	831	*Hafnia alvei*<br>*Raoultella ornithinolytica*<br>*Enterococcus faecalis*<br>*Enterococcus faecium*<br>*Lactobacillus plantarum*	8.77
Brie	France	-	-	6.45	20.0	-	-	-	-	26.5	Not identified	8.49
Brie	France	-	-	6.61	15.1	-	-	-	-	21.7	*Raoultella ornithinolytica*<br>*Serratia marcescens*<br>*Lacticaseibacillus paracasei*	9.15
Brie	France	-	-	-	-	-	-	8.29	-	8.29	Not identified	7.85
Brie de Meaux	France	-	-	331	1137	-	9.09	-	-	1,477	*Hafnia alvei*<br>*Enterococcus faecalis*	9.43

										Enterococcus malodoratus<br>*Lactobacillus plantarum*	
Brie petit	France	-	-	7.44	10.8	-	-	-	-	18.2	*Raoultella ornithinolytica*<br>*Lactococcus lactis*	9.23
Munster la Sapiniere	France	-	-	-	-	68.5	128	6.48	-	203	*Lelliottia amnigena*<br>*Providencia rettgeri*<br>*Enterococcus faecalis*<br>*Enterococcus faecium*<br>*Lactococcus lactis*	9.11
Munster la Sapiniere	France	-	-	-	-	9.43	186	9.62	-	205	*Enterococcus faecalis*<br>*Lactococcus lactis*	9.60
Munster Grome	France	-	-	37.7	8.81	-	9.19	-	-	55.7	*Lelliottia amnigena*<br>*Lactococcus lactis*	9.38
La Brique	France	-	-	-	70.6	-	-	-	-	70.6	*Hafnia alvei*<br>*Lactococcus lactis*	9.20
La Brique	France	-	-	-	-	-	-	-	-	-	*Lactococcus lactis*	8.88
Snack a la francaise	France	-	-	-	-	-	-	-	-	-	Not identified	8.60
Snack	France	-	-	-	-	-	-	-	-	-	*Lelliottia amnigena*<br>*Enterococcus gilvus*	8.71
Soft cheese	Germany	-	-	-	7.12	-	-	7.66	-	14.8	*Serratia liquefaciens*<br>*Enterococcus faecalis*	7.18
Soft cheese	Poland	-	-	-	-	-	-	8.66	-	8.66	*Lactococcus lactis*<br>*Leuconostoc pseudomesenteroides*	9.04
Caprice des Dieux	France	-	-	-	-	-	-	7.74	-	7.74	*Lactococcus lactis*	7.08
Carre de l'Est	France	-	-	-	35.2	-	-	9.06	-	44.3	*Hafnia alvei*<br>*Lactococcus lactis*	9.08
Chevre St. Maure	France	-	-	-	-	10.7	-	9.77	-	24.5	*Lactococcus lactis*	9.46
Coulommiers	France	-	-	110	828	-	-	8.96	-	947	*Hafnia alvei*<br>*Enterococcus faecium*<br>*Leuconostoc pseudomesenteroides*<br>*Leuconostoc mesenteroides*	8.58
Fromage de Bretange	France	-	-	6.06	-	-	-	-	-	6.06	*Enterococcus faecalis*<br>*Leuconostoc pseudomesenteroides*<br>*Lactococcus lactis*	8.56
Fromage de Normandie	France	-	-	-	-	-	-	-	-	-	*Enterococcus faecalis*<br>*Lactococcus lactis*	8.65
Fromage du Calvados	France	-	-	-	-	-	-	-	-	-	*Lactococcus lactis*	8.88
La Rustigue Carre	France	-	-	-	6.37	-	-	-	-	6.37	*Hafnia alvei*<br>*Lactococcus lactis*	9.15
Langres	France	-	-	-	14.8	-	-	11.6	6.52	33.0	*Leuconostoc mesenteroides*	8.79
Pave d'Affiniois	France	-	-	-	-	-	-	11.9	-	11.9	*Enterococcus faecalis*<br>*Enterococcus faecium*	8.85
Saint Albray	France	-	-	-	-	-	-	-	-	-	Not identified	8.77
Saint Felicien	France	-	-	19.0	265	-	-	-	-	284	*Hafnia alvei*<br>*Enterococcus devriesei*<br>*Enterococcus malodoratus*<br>*Lactococcus lactis*	9.11
Blue-veined chesses (n = 44)												
Gorgonzola	Italy	-	-	-	-	101	-	7.20	-	108	*Enterococcus faecium*<br>*Serratia liquefaciens*	8.20
Gorgonzola	Italy	-	-	7.57	-	91.1	-	-	-	98.7	*Enterococcus faecalis*	7.30
Gorgonzola	Italy	-	8.37	-	-	127	360	8.25	-	504	*Enterococcus faecium*	8.04

Gorgonzola	Italy	-	-	-	-	101	-	9.39	-	110	*Enterococcus faecium*	8.08
Gorgonzola	Italy	-	-	-	-	246	5.88	8.48	-	260	*Enterococcus faecium*<br>*Leuconostoc pseudomesenteroides*	7.28
Gorgonzola	Italy	-	-	-	-	179	411	-	-	590	*Enterococcus faecium*<br>*Lacticaseibacillus paracasei*	8.68
Gorgonzola	Italy	-	-	-	-	-	-	7.89	-	7.89	*Enterococcus faecium*	7.78
Gorgonzola	Italy	-	35.8	-	-	6.23	583	-	-	625	*Enterococcus faecium*<br>*Serratia liquefaciens*	7.89
Gorgonzola	Italy	-	-	-	-	298	-	-	-	298	*Lactobacillus delbruecki*	7.11
Gorgonzola	Italy	-	-	-	30.0	236	646	-	-	912	*Lacticaseibacillus paracasei*<br>*Lactobacillus delbruecki*<br>*Serratia liquefaciens*	8.97
Gorgonzola	Italy	-	-	-	-	342	54.9	-	-	397	*Lactobacillus delbruecki*	8.00
Gorgonzola	Italy	-	-	-	-	-	33.7	6.99	-	40.7	*Serratia liquefaciens*	7.74
Gorgonzola	Italy	-	-	-	-	194	-	-	-	194	Not identified	8.11
Gorgonzola	Italy	-	-	-	-	-	68.5	-	-	68.5	Not identified	7.96
Gorgonzola	Italy	-	-	-	-	127	-	-	-	127	*Enterococcus faecium*	7.23
Roquefort	France	-	-	-	6.12	-	21.6	6.97	-	34.7	*Enterococcus faecalis*<br>*Enterococcus hirae*<br>*Leuconostoc mesenteroides*	8.51
Roquefort	France	-	-	-	-	-	-	-	-	-	*Enterococcus faecium*<br>*Enterococcus faecalis*<br>*Enterococcus hirae*<br>*Lacticaseibacillus paracasei*<br>*Leuconostoc mesenteroides*	7.28
Roquefort	France	-	-	-	-	-	-	-	-	-	*Enterococcus faecalis*<br>*Enterococcus faecium*<br>*Enterococcus hirae*<br>*Leuconostoc mesenteroides*	8.11
Roquefort	France	-	-	-	-	-	-	8.76	-	8.76	*Enterococcus faecalis*<br>*Enterococcus faecium*<br>*Enterococcus hirae*<br>*Lactococcus lactis*<br>*Leuconostoc mesenteroides*	8.74
Roquefort	France	-	-	-	-	-	101	-	-	101	*Enterococcus faecalis*<br>*Enterococcus hirae*<br>*Leuconostoc mesenteroides*	8.00
Fourme d'ambert	France	-	-	-	-	-	-	-	-	-	*Enterococcus faecalis*<br>*Leuconostoc mesenteroides*	8.08
Fourme d'ambert	France	-	15.7	-	-	-	-	13.8	-	29.5	Not identified	8.26
Fourme d'ambert	France	-	-	-	-	-	-	11.1	-	11.1	*Leuconostoc pseudomesenteroides*	8.75
Fourme d'ambert	France	-	-	-	-	-	-	10.1	-	10.1	*Enterococcus faecalis*	8.49
Bleu d'auvergne	France	-	-	-	-	-	-	9.52	-	9.52	*Leuconostoc mesenteroides*	8.40
Bleu d'auvergne	France	-	-	-	-	-	-	-	-	-	*Leuconostoc mesenteroides*	8.58
Bleu d'auvergne	France	-	-	-	-	-	-	6.43	-	6.43	Not identified	7.78
Danablu	Denmark	-	-	-	-	-	8.06	8.37	-	16.4	*Leuconostoc mesenteroides*	8.15
Danablu	Denmark	-	-	-	-	-	-	-	-	-	Not identified	7.36
Danablu	Denmark	-	11.1	-	-	-	-	-	-	11.1	Not identified	6.96
Lazur	Poland	-	-	-	-	-	-	11.1	-	11.1	*Leuconostoc pseudomesenteroides*	8.23
Lazur	Poland	-	-	-	-	-	-	14.8	-	14.8	Not identified	7.45
Lazur	Poland	-	-	-	-	-	-	-	-	-	Not identified	7.00
Bavaria blue	Germany	-	9.76	-	-	-	-	6.67	-	16.4	*Enterococcus faecalis*<br>*Leuconostoc mesenteroides*	9.00

Bavaria blue	Germany	-	-	-	-	-	16.8	-	-	16.8	*Enterococcus faecium*<br>*Enterococcus malodoratus*<br>*Enterococcus gilvus*<br>*Leuconostoc mesenteroides*	8.26
Cambozola	Germany	-	-	-	-	-	-	-	-	-	*Lacticaseibacillus paracasei*	9.20
Cambozola	Germany	-	-	-	-	-	-	-	-	-	Not identified	9.04
Kamieniogórski	Poland	-	-	-	-	-	133	16,5	-	150	*Enterococcus faecalis*	8.68
Kamieniogórski	Poland	-	-	-	-	-	51.6	15.0	-	66.6	*Enterococcus faecalis*<br>*Lactobacillus curvatus*	8.20
Bleu des causses	France	-	-	-	-	-	-	10.4	-	10.4	*Leuconostoc mesenteroides*<br>*Enterococcus faecalis*	8.56
Dorblu	Germany	-	-	-	-	-	-	11.6	-	11.6	*Leuconostoc pseudomesenteroides*	8.04
Le Bleu	France	-	-	-	-	-	-	8.97	-	8.97	Not identified	8.23
Saint Agur	France	-	-	5.74	-	-	-	13.9	-	19.6	Not identified	8.86
Stilton	U.K.	-	-	-	-	-	-	6.91	-	6.91	*Lactobacillus plantarum*	8.18
Semi-hard cheeses (n = 14)												
Raclette	France	-	19.7	-	8.14	7.14	223	-	-	258	*Enterococcus malodoratus*<br>*Lactococcus lactis*	9.34
Raclette	France	-	-	-	18.4	-	40.3	-	-	58.7	*Lactococcus lactis*	8.26
Raclette	France	-	-	-	7.04	68.5	32.2	6.58	-	114	*Providencia heimbachae*<br>*Enterococcus faecalis*<br>*Enterococcus malodoratus*<br>*Lactococcus lactis*	8.76
Raclette	France	-	36.1	-	-	-	147	-	-	183	*Enterococcus malodoratus*<br>*Lactobacillus curvatus*<br>*Lactococcus lactis*	8.76
Tomme de Montagne	France	-	-	-	-	-	-	-	-	-	*Lactococcus lactis*	9.327
Tomme de Montagne	France	-	-	7.06	22.3	-	-	-	-	29.4	*Lactococcus lactis*	7.52
Breton	France	-	-	5.87	19.9	-	-	21.3	-	47.1	*Hafnia alvei*<br>*Serratia liquefaciens*<br>*Enterococcus faecalis*<br>*Lactococcus lactis*	8.51
Caciotta with chili and arugula	Italy	-	16.1	21.6	-	30.7	89.3	-	-	158	*Escherichia coli*<br>*Enterococcus faecium*<br>*Lacticaseibacillus paracasei*<br>*Lactobacillus curvatus*<br>*Lacticaseibacillus rhamnosus*	9.00
Memel with basil	Lithuania	-	-	-	-	-	32.3	-	-	32.3	*Lactobacillus curvatus*	7.86
Mimolette	Holand	-	-	-	-	-	-	9.93	-	9.93	Not identified	5.52
Morbier	France	-	-	304	506	112	334	-	-	1,256	*Hafnia alvei*<br>*Serratia liquefaciens*<br>*Enterococcus faecalis*<br>*Lacticaseibacillus paracasei*<br>*Leuconostoc mesenteroides*<br>*Lactococcus lactis*	8.99
Queijo Curado Mistura	Portugal	-	-	-	-	-	-	-	-	-	*Lactococcus lactis*	8.72
Spanish Matured Goats cheese	Spain	-	-	-	-	-	-	-	-	-	*Lactococcus lactis*	7.74
Tricolore	Netherland s	-	-	-	-	-	-	13.2	-	13.2	*Lactococcus lactis*	6.71
Hard cheeses (n = 29)												
Parmigiano Reggiano	Italy	-	-	-	-	74.8	6.99	-	-	81.8	Not identified	< 5.00

Parmigiano Reggiano	Italy	-	-	-	-	53.0	11.4	-	-	64.4	*Lacticaseibacillus paracasei*	2.87
Parmigiano Reggiano	Italy	-	-	-	-	137	-	-	-	137	*Pediococcus acidilactici*	5.66
Parmigiano Reggiano	Italy	-	-	-	-	-	-	-	-	-	*Lacticaseibacillus paracasei*	4.56
Parmigiano Reggiano	Italy	-	-	-	-	163	-	-	-	163	*Lacticaseibacillus paracasei*	5.97
Parmigiano Reggiano	Italy	-	-	-	-	65.1	-	-	-	65.1	*Lacticaseibacillus paracasei*	3.15
Parmigiano Reggiano	Italy	-	-	-	-	59.9	-	-	-	59.9	Not identified	< 1.00
Grana Padano	Italy	-	-	-	-	23.1	-	-	-	23.1	*Lacticaseibacillus rhamnosus*	4.30
Grana Padano	Italy	-	-	-	-	32.0	5.62	-	-	37.6	*Lacticaseibacillus paracasei*	6.08
Grana Padano	Italy	-	-	-	-	67.6	-	-	-	67.6	*Lactobacillus plantarum*<br>*Lacticaseibacillus rhamnosus*<br>*Lacticaseibacillus paracasei*	3.00
Grana Padano	Italy	-	-	-	-	152	-	-	-	152	*Lacticaseibacillus paracasei*	4.38
Grana Padano	Italy	-	-	-	-	49.1	-	-	-	49.1	*Lacticaseibacillus rhamnosus*<br>*Lacticaseibacillus paracasei*<br>*Pediococcus acidilactici*	5.76
Grana Padano	Italy	-	-	-	-	37.3	-	-	-	37.3	*Lacticaseibacillus rhamnosus*<br>*Pediococcus acidilactici*	4.56
Cheddar	UK	-	-	-	-	-	-	-	-	-	*Lacticaseibacillus rhamnosus*<br>*Lacticaseibacillus paracasei*	6.75
Cheddar	UK	-	-	-	-	-	-	-	-	-	*Lactococcus lactis*<br>*Lacticaseibacillus paracasei*	6.75
Grated dehydrated cheese (Gratino)	Italy	-	-	22.3	180	91.8	29.9	-	-	324	*Enterococcus faecium*<br>*Lacticaseibacillus paracasei*	4.32
Grated dehydrated cheese (Gratino)	Italy	-	-	117	138	87.1	262	-	-	604	*Pediococcus acidilactici*	4.41
Fromagia	Italy	-	-	-	-	-	-	-	-	-	*Lacticaseibacillus paracasei*	5.28
Corregio	Poland	-	-	-	-	-	81.6	6.52	-	88.1	*Lacticaseibacillus paracasei*	7.04
Graviera Kritis	Greece	-	-	-	-	-	14.2	-	-	14.2	*Enterococcus faecalis*<br>*Enterococcus faecium*<br>*Lacticaseibacillus paracasei*	6.56
Gruyere	Switzerland	-	-	-	17.0	-	-	12.4	7.28	36.7	*Lacticaseibacillus paracasei*	6.30
Monte Veronese	Italy	-	-	-	10.7	-	35.0	-	-	45.7	*Enterococcus faecalis*<br>*Hafnia alvei*<br>*Lacticaseibacillus paracasei*	8.58
Old Amsterdam	Netherlands	-	-	-	-	-	-	-	-	-	Not identified	2.70
Oscypek	Poland	-	-	-	-	-	-	-	-	-	Not identified	2.34
Percorino Romano	Italy	-	-	-	9.82	8.24	5.62	-	-	23.7	*Enterococcus faecalis*<br>*Lacticaseibacillus paracasei*	7.48
Queso de Cabra	Spain	-	-	-	-	-	-	-	-	-	*Lactococcus lactis*	8.00
Queso de Manchego	Spain	-	-	-	-	-	-	-	-	-	*Lactococcus lactis*<br>*Lactobacillus curvatus*	7.53
Queso Iberico	Spain	-	-	-	-	-	-	-	-	-	*Lacticaseibacillus paracasei*<br>*Lactobacillus curvatus*	6.67

Scharfer Paul	Switzerland	-	-	17.0	68.2	-	-	-	-	85.2	*Lactococcus lactis*	8.65

* Total number of microorganisms (log10 CFU/g).
